# Perceived barriers to pharmacist engagement in adverse drug event prevention activities in Ghana using semi-structured interview

**DOI:** 10.1186/s12913-015-1031-9

**Published:** 2015-09-07

**Authors:** Franklin Acheampong, Berko Panyin Anto

**Affiliations:** Pharmacy Department, Korle Bu Teaching Hospital, Accra, Ghana; Department of Clinical & Social Pharmacy, Faculty of Pharmacy & Pharmaceutical sciences, Kwame Nkrumah University of Science and Technology, Kumasi, Ghana

## Abstract

**Background:**

Pharmacist involvement in the prevention of medication errors is well documented. One such method, the process by which hospital pharmacists undertake these clinical interventions needs to be described and documented. The perceived barriers to pharmacists succeeding in getting their recommendations accepted could inform future safety strategy development. This study was therefore to trace the typical process involved and explore the perceived barriers to pharmacists’ medication safety efforts.

**Methods:**

This study involved a retrospective evaluation of routine clinical interventions collected at a tertiary hospital in Ghana over 23 months. A sample of pharmacists who had submitted these reports were then interviewed.

**Results:**

The interventions made related to drug therapy changes (76.0 %), monitoring (13.0 %), communication (5.4 %), counselling (5.0 %) and adverse drug events (0.6 %). More than 90 % of interventions were accepted. The results also showed that undertaking clinical interventions by pharmacists followed a sequential order with two interlinked subprocesses: *Problem Identification and Problem Handling*. In identifying the problem, as much information needed to be gathered, clinical issues identified and then the problems prioritised. During the problem handling stage, detailed assessment was made which led to the development of a pharmaceutical plan. The plan was then implemented and monitored to ensure appropriateness of desired outcomes.

The main barrier mentioned by pharmacist related to the discrepant attitudes of doctors/nurses. The other barriers encountered during these tasks related to workload, and inadequate clinical knowledge. The attitudes were characterised by conflicts and egos resulting from differences in status/authority, responsibilities, and training.

**Conclusions:**

Though the majority of recommendations from pharmacists were accepted, the main barrier to hospital pharmacist engagement in medication error prevention activities related to discrepant attitudes of doctors and nurses. Proper initiation and maintenance of collaborative working relationship in hospitals is desired between the healthcare team members to benefit from the medication safety services of hospital pharmacists.

## Background

The clinical values of pharmacists’ intervention and its positive contribution to the quality of pharmacotherapy have been confirmed in literature [1–3]. For example, in an acute care geriatric unit, 76 interventions were made in 3-month period in pharmacotherapy areas that included drug selection, dosing, changes in therapy, and medication reconciliation [4]. The clinical role of pharmacists involves preventing, identifying, and resolving medication errors. The involvement of pharmacists to reduce the risk and harm of medication errors is thus complementary to many recent automated or mechanical improvements relating to prescribing, dispensing and administering medications. Pharmacist interventions have been shown to positively affect clinical health outcomes such as morbidity and adverse drug events [2, 5]. Though the majority of medication related errors do not reach the patient due to pharmacists, nurses and other healthcare professionals, their potential to cause morbidity and mortality is significant [6]. Undoubtedly, medication errors that have been prevented need to be studied. When interventions are made, it means an accident sequence was initiated and then by the actions of the individual, team or organisation it was prevented from having negative consequences. Documentation of the interventions is important for justifying pharmacists’ services to the patient, healthcare managers and providers, patient care takers, and to strengthen the profession [7]. Usually, there are some barriers to undertaking this very important clinical function of pharmacist. Clinical interventions also go through well-thought processes that would inform practitioners and assist in planning specific roles for pharmacists along the process nodes.

The objectives of the study were:

- Evaluate clinical intervention reports submitted by hospital pharmacists
- Describe the processes involved in performing clinical interventions by hospital pharmacists.
- Explore the perceived barriers encountered by pharmacists in their medication error prevention activities

## Method

### Study Setting

The study took place at Korle Bu Teaching hospital which is a 2000 bed tertiary teaching hospital located in the capital city of Ghana. At the time of the study, the hospital had about 80 pharmacists. The main pharmacy services provided in the hospital were dispensing, clinical services, drug information, research and small scale manufacturing. There were about 30 pharmacists who actively undertook clinical duties across the various wards of the hospital together with other health care professionals.

### Study design

**Fig. 1:**
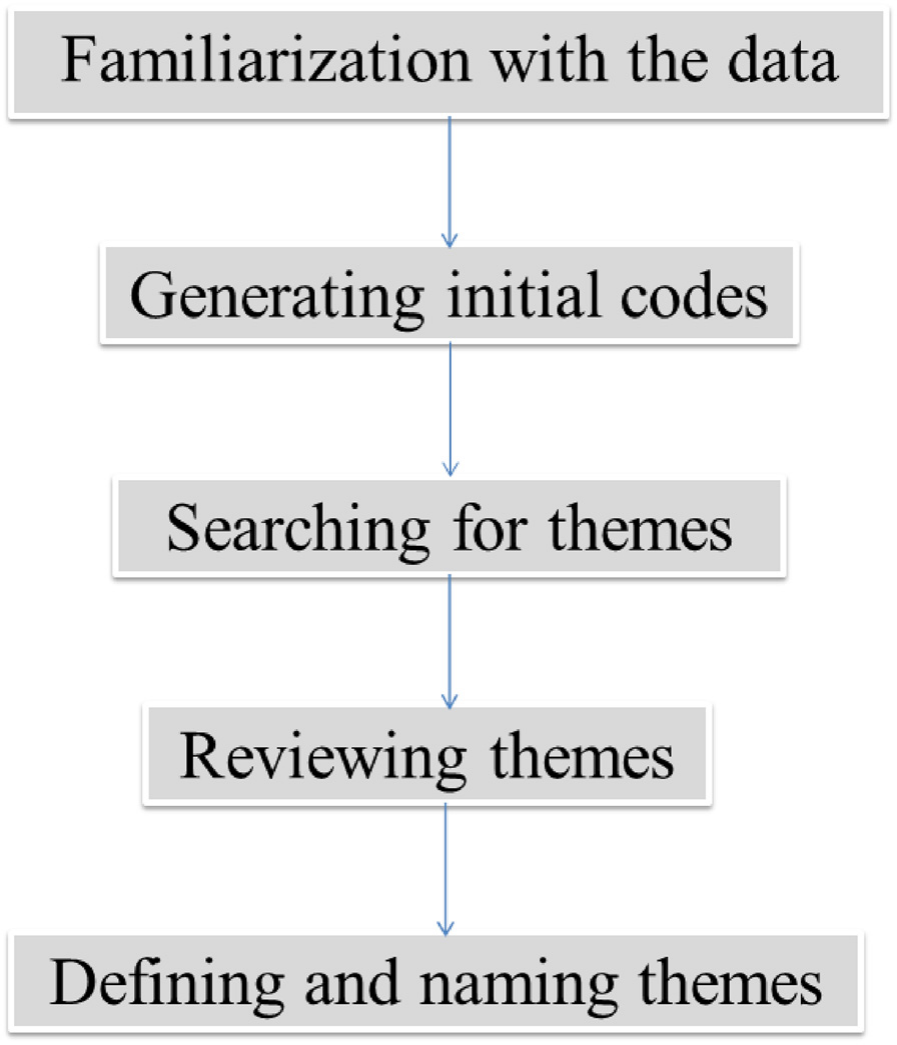
Stages of Thematic Content analysis of interviews recommended by Braun and Clarke adopted from ‘Identifying the Latent Failures Underpinning Medication Administration Errors: An Exploratory Study’ by Lawton et al. [36]

The study involved a retrospective evaluation of clinical intervention reports of hospital pharmacists who were engaged in direct patient care. This was almost immediately followed by key informant interviews with sampled pharmacists whose clinical intervention reports had been evaluated. Interview techniques are useful in gaining rich insight into a subject matter. Researchers used interviews to explore the possible causes of different types of medication errors [8–11]. The interview process probes and provides detailed information as well as other useful information that the researcher had not considered prior to the start of a study. Authors have pointed to the utility of interview techniques in gaining rich information regarding patient safety incidents [12, 13]. Interviews were then subjected to thematic content analysis. Braun and Clarke [14] developed the five stages of content analysis which has been described in Fig. 1.

### Data collection

In determining the eligible participants for the key informant interview, the outcome of the review of intervention reports was used. Evaluation of the intervention reports revealed that 24 pharmacists reported clinical interventions during the period January 2011 to December 2013. As at the time of the interview, 5 pharmacists were not available to be interviewed. Additionally, 2 pharmacists were no longer actively performing clinical duties. Seventeen pharmacists were finally declared eligible to participate in the study and they were all invited with a letter. In addition to the letter, information sheets and consent forms were added. The interviewees had varying degree of experience, in terms of years of practice and professional grade. Interviewees who had spent less than 5 years in practice submitted slightly higher intervention reports. Two major categories were sought from interviewees’ response to questions which focused on how participants thought through the process they used and the challenges experienced in undertaking interventions.

With the aid of semi-structured guide, the interviews were conducted in the pharmacists’ offices between 11^th^ March and 8^th^ April 2014. The interview lasted between 30 and 35 min. Interviews were recorded with an audio recorder with the permission of each participant. Interviews were then transcribed verbatim. The transcripts of the interviews were coded to maintain confidentiality of interviewees and then subjected to content analysis to draw out common themes.

Ethical approval was obtained from the Ethical and Protocol Review Committee of the University of Ghana Medical School.

## Results

Twenty four pharmacists made 1019 clinical interventions in 448 handwritten reports. The interventions related to drug therapy changes (76.0 %), monitoring (13.0 %), communication (5.4 %), counselling (5.0 %) and adverse drug events (0.6 %). More than 90 % of interventions and recommendations by pharmacists were accepted and implemented whilst over 70 % of the interventions involved drug regimen change.

A total of 17 pharmacists were invited to participate in the interviews. Out of this, 12 pharmacists (70.6 %) agreed to be interviewed and completed consent forms. The characteristics of the interviewees are summarised in Table 1.

**Table 1:** Characteristics of interviewees (*n* = 12)

Item	Number (*n* = 12)	Number of reports submitted (*n* = 448)
Years of practice		
<5	5	227
5-10	2	69
>10	5	152
Grade of pharmacist		
Pharmacist	4	198
Senior Pharmacist	3	98
Principal Pharmacist	3	71
Specialist Pharmacist	2	81
Median number of intervention reports per pharmacist (*n* = 24) (min-max)	22 (16–28)	

### Process sequence

Respondents described the process used in performing clinical interventions. Undertaking clinical interventions followed a sequential order which is made up of two linked subprocesses. The two subprocesses were *Problem Identification and Problem Handling*. In identifying the problem, as much information needed to be gathered, clinical issues identified and then the problems prioritised. During the problem handling stage, detailed assessment was made which led to the development of a pharmaceutical plan. The plan was then implemented and monitored to ensure appropriateness of desired outcomes.

**Fig. 2:**
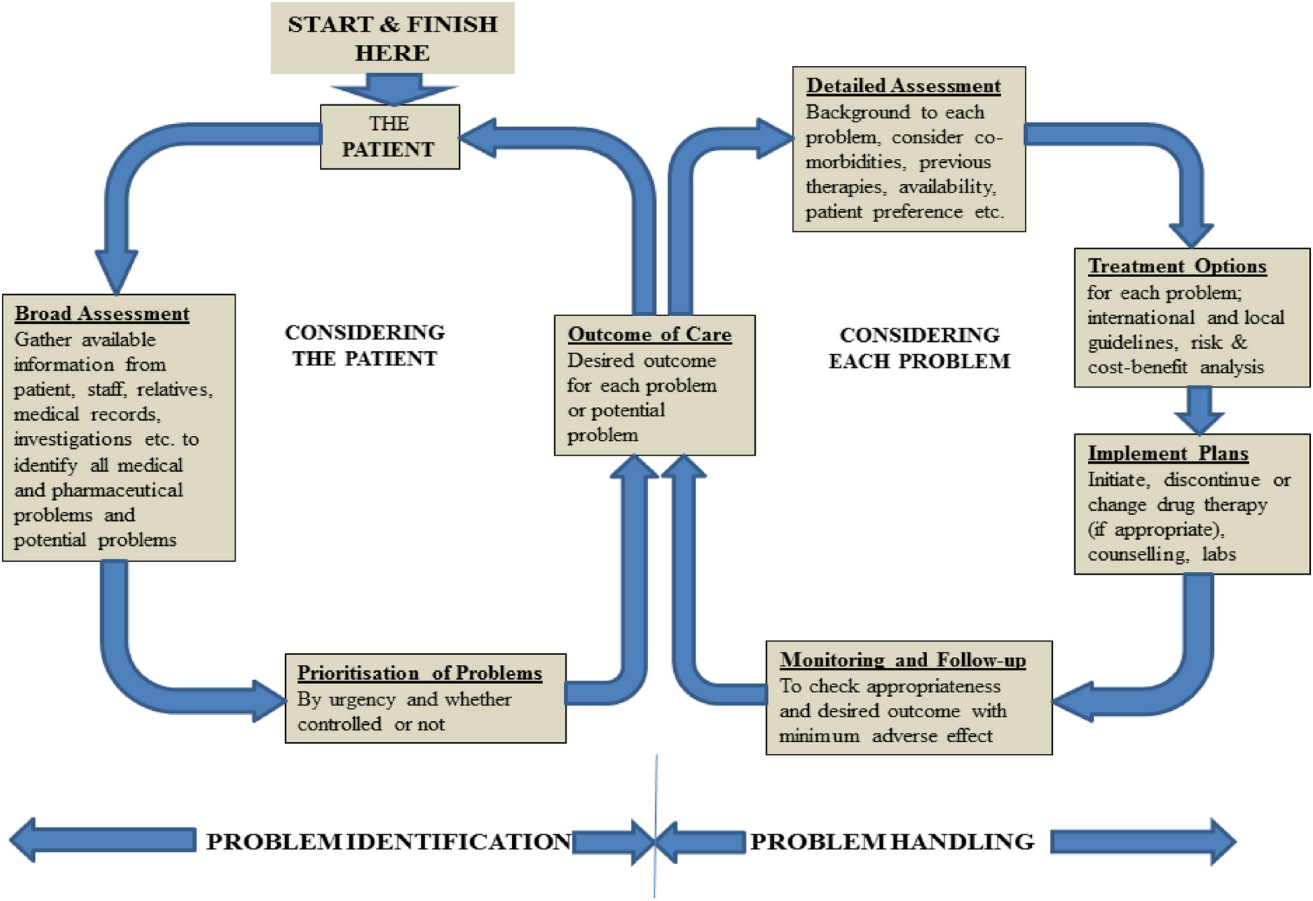
Schematic representation of clinical interventions process

Figure 2 shows a schematic representation of the various steps taken by participants in their clinical intervention process. The following are some excerpts:

#### Gathering of information

Interviewees mentioned that they gather relevant information by reviewing patients’ medical notes, talking to patients, their relatives, nurses or physicians. Two participants described how they gather information on patients:

*“In obtaining relevant information, we review patients’ medical and biomedical records like the LFT’s. An example is a patient may be on a correct dose or frequency of a drug but it maybe contraindicated because the LFT’s maybe deranged so there comes the need for varying dose frequencies for that particular patient.” (Participant004)*

*“Participating in ward rounds with other healthcare professionals also provide pharmacists the opportunity to discuss the patients in details and thereby obtain first-hand patient information.” (Participant 001)*

#### Identification of issues

Respondents then identified pharmaceutical issues that required interventions. The issues mentioned were medication errors. The issues formed the basis for the interventions. They included prescribing errors, dispensing errors, medication administration errors, monitoring requirements, counselling requirement, and adverse drug reaction reporting. A quote illustrates this point:

*“You look at the diagnosis, look at the vital signs, relate it to the medications and find out if there is any problems. You watch out for drug-drug interactions, adverse effects related to the medication. We have the opportunity to look at lab reports and other investigations. We look at other parameters like urine output, blood sugar, patient weight and link it to patients’ medication too.” (Participant 011)*

#### Development of pharmaceutical plan

After the problems had been identified, a plan was designed. The plan involved the actions to be taken to resolve the problem. This was illustrated by respondents.

*“We start by assessing the identified problems, using the background knowledge in drug therapy. After assessing the problems we plan on how the changes can be made. This involves consulting the medical practitioner in charge of the said patient.” (Participant 001)*

#### Communicating/Implementation of plan

After a plan has been developed, it is either communicated or implemented. Pharmacist compared the plan with local, regional or international protocols. These plans were patient centred as summarised by a respondent:

*“Usually once you identify a problem, you need to draw the attention of the other healthcare professionals about the problem identified and obviously help find a solution to it. Sometimes you will get queries demanding a defense to your point, most of which is done verbally.” (Participant 005)*

#### Monitoring or feedback

Respondents mentioned that this stage of the process was to compare results with desired outcomes and ensured minimum side effects. Two excerpts are provided below:

*“When all is said and done, you cannot go to sleep. You have to plan to monitor the interventions made. You will need feedback of a sort. Say, clinical outcome etc.” (Participant 002)*

*“After that, we evaluate the changes.” (Participant 005)*

### Barriers to performing clinical interventions

All respondents stated that they experienced one challenge or another. Three secondary categories were identified as potential barriers to performing interventions. They included workload, lack of clinical knowledge and attitudes of doctors and nurses.

- Workload

All the interviewees mentioned that workload was a challenge they encountered daily. They commented that they had to perform other roles like dispensing, stock management etc. in addition to clinical roles.

*“But sometimes the workload becomes too much to bear that you even want to run away even though I think its fulfilling especially when you see a patient get better because of an intervention you made*.” *(Participant 003)*

*“Because of time factor, it is very difficult for me to go on ward rounds to identify patient care issues and then make the necessary interventions. The same person is managing the pharmacy, attending meetings and so on*.” *(Participant 004)*

b) Lack of adequate clinical knowledge,

Adequate clinical knowledge is essential to undertake effective clinical roles. Ten out of the 12 participants agreed that another major barrier was lack of clinical knowledge. Participants supported the need for postgraduate degree qualification for pharmacists to undertake this clinical role effectively. Two participants illustrate this:

*“Sometimes you feel inadequate, in terms of clinical knowledge and extra training will be helpful. The first degree is not adequate because it makes it difficult for you to make contributions into challenging issues.” (Participant 002)*

*“It’s sometimes intimidating because probably it’s a consultant you are going to see and you are scared you don’t have your facts right. And the doctors come to the wards in bunches and we go single handedly.” (Participant 010)*

Participants also mentioned the lack of specialist training. Participants believe that specialist training will contribute to enhancing clinical knowledge.

*“What I am doing is general clinical pharmacy and I feel inadequate sometimes because the cases are specialist cases. If I were to specialize in a particular area for example, cardiology, I think I would perform better. And also it will add more width to my work and people will appreciate my input more because I will have more knowledge in a particular field.” (Participant 011)*

In contrast, a participant suggested that Pharm D qualification would prepare pharmacy graduates to better perform clinical roles immediately.

*“I preferred Pharm D. training program which seeks to make the pharmacist very capable of providing clinical care at the point of completion of the course.” (Participant 006)*

c) Doctors’ and nurses’ attitudes

In the clinical settings, pharmacists work closely with other healthcare professionals, especially doctors and nurses. Participants mentioned that another major barrier to their clinical role was the attitudes and perceptions of doctors and nurses. The statements below summarise the point:

*“There is this attitude of looking down on others by doctors because of professional differences or backgrounds, and they probably feel that one is questioning their authority. Doctors, especially feel superior because they are ultimately responsible for the patient; they overuse that. It is purely an issue of ego.” (Participant 012)*

*“A typical example was when a prescription of vitamin C 3000 mg was written for a child of 2 years by a house officer. I quickly wrote a note to indicate the error in the prescription. He sent it back insisting that it be given. He was particularly rude. I went to him personally to inquire reasons he was insisting that dose be given to a child of 2 years. It became a rather tense and confrontational situation and I told him that unless he changed the prescription I wasn’t going to dispense the drug.” (Participant 005)*

Some doctors and nurses are ignorant of the clinical competence of pharmacists and these have the potential of influencing their attitudes toward pharmacists. Below are some quotes from what they said:

*“There is this mentality that doctors are to take care of patients and pharmacists are to look after drugs without any consideration to how they were used at all.” (Participant 011)*

*“There is always that doubt about the competence of pharmacist by other professionals. Doctors are trained that one has to prove his worth to be able to play a role in patient management so it is only natural that there will be some difficulty in accepting another person who was not trained the same way as doctors.” (Participant 010)*

*“There has to be a form of platform that seeks to inform them of our abilities and readiness play clinical roles and not just our traditional roles of dispensing, manufacturing and the rest. We have evidence. For example, when we got actively involved in HIV care, our patients are well educated and we are achieving 90 % adherence and most of our patients are doing well.” (Participant 012)*

*“People even think that when it comes to hospital, its doctor or nurse, finish!. Even though doctors and nurses appreciate our work they seem to forget themselves sometimes and forget our clinical roles.” (Participant 003)*

Lack of proper communication skills can also create unfavourable working relationships with others and lead to improper attitudes.

*“Unfortunately the premise of interventions is more like corrective and if you don’t have the right skills to make recommendations, they will become defensive. No one wants to be told they have done mistakes, especially doctors.” (Participant 011)*

*“Your approach, communication is very important, if you don’t approach properly, then the other one thinks you are discrediting his role or questioning his integrity or knowledge, there must be rapport such that the person would accept your input.” (Participant 002)*

Lack of cooperation was also mentioned by participants as a barrier. Interviewees perceived that it sometimes appeared as if doctors and nurses were protecting their turf. The following illustrates the assertion:

*“It is purely the lack of collaboration and cooperation. If the system does encourage people to meet and work together in the interest of your patient, then it’s easier for everybody to cooperate.” (Participant 007)*

*“There is the mentality that doctors and nurses are supposed to be taking care of patients and we are supposed to be taking care of products without consideration to how they were used. The thought of trying to be doctors by adding patient care to our responsibilities will lead to them asking for the same benefits they receive.” (Participant 012)*

## Discussion

The study evaluated the clinical intervention reports submitted by pharmacists working in a tertiary hospital and explored the process involved and the potential barriers to pharmacist clinical interventions. The pharmacists identified medication errors in the management of patients and made interventions to prevent these errors from reaching patients. Twenty four pharmacists made 1019 clinical interventions in 448 handwritten reports. Majority of the interventions related to drug therapy changes.

The most frequently reported medication errors found in our study were medication regimen change and originated from medication prescribing. Studies have reported prescribing errors as a major contributor to patient harm in hospitals [15–19]. This finding is consistent with findings from other studies conducted in clinical centres [16], tertiary [20], hospital inpatient [15] and ambulatory care settings [21]. More than 90 % of interventions and recommendations by pharmacists were accepted and implemented.

Pharmacists are in an ideal position to provide ongoing medication therapy management services for their patients. Error recovery is one of such important roles. Pharmacist interventions have been shown to positively affect clinical health outcomes such as morbidity and adverse drug events [2, 5]. There are however barriers to optimizing such a useful medication safety strategy. When these barriers interfere with the pharmacist’s ability to perform interventions, serious errors may reach the patient.

Pharmacists clinical intervention in this study followed a sequential process which was cyclical in nature (see Fig. 2). The attributes of the process compares with the process of providing pharmaceutical care which has been described as a continuous quality improvement process for the use of medications [22]. Hepler and Strand described pharmaceutical care as the responsible provision of medication therapy for the purpose of achieving definite outcomes that improve or maintain a patient’s quality of life [23]. A fundamental element in optimising patient outcomes is the routine addition of ongoing monitoring to the medication use process through the participation in patient care processes [24]. The process as described begins by assessing the patient. This will involve gathering information about the patient records. The patient’s record will include medical history, past and present drug therapies, social, family and drug allergy history. The records are then reviewed to identify the potential medical and pharmaceutical problems. These identified problems are then prioritised based on urgency and importance. In this prioritisation stage, the desired outcomes for each problem are set out. This is done to guide the formulation of individualised intervention. Subsequently, a detailed assessment, making particular reference to co-morbidities, patient specific characteristics etc., is made. The next stage will involve deciding which actions to take and how these actions are implemented. This is done by referring to acceptable local or international standards, protocols, guidelines and formulae. This is also the stage where cost-benefit analyses are considered [25]. The plans are then implemented. Strategies are then put in place to monitor and evaluate the intervention made.

The barriers to clinical interventions mentioned by interviewees related to workload, inadequate clinical knowledge and attitudes of doctors and nurses. Workload negatively affects pharmacists’ performance on various activities undertaken at various settings [26–28]. Workload has been known as potential causes of medication errors [9]. In a study, pharmacist’s willingness or ability to intervene in the case of prescription problems decreased as the volume of prescriptions dispensed increased [29]. Leape et al. [30] also found that workload was a contributory factor to adverse drug events and recommended reducing workload in hospitals.

Pharmacists mentioned that their inadequate clinical knowledge affected their performance. Appropriate knowledge about therapeutics will assist pharmacists in being seen as partners in the clinical management of patients. Physician’s familiarity with and respect for a pharmacist’s clinical abilities could support his or her willingness to accept a pharmacist’s input. Pharmacists with the requisite clinical training and professional education are positioned to prevent errors and help in patient management because of their knowledge and skills in medication therapy and their accessibility to patients [31]. Pharmacists are able to blend a caring orientation with specialized therapeutic knowledge, experience, and judgment for the purpose of ensuring optimal patient outcomes [32].

The negative attitudes of doctors and nurses towards pharmacist were identified as potential barriers. Discrepant attitudes about healthcare teamwork characterised by conflicts and egos resulted from differences in status/authority, responsibilities, training, and nurse and doctor cultures [33]. Hughes and McCann [34] found that doctors did not appreciate pharmacists in healthcare. In this study, though majority of the recommendations were accepted and implemented, pharmacists still mentioned that they frequently experienced unfavourable attitudes from doctors and nurses on the wards. The role of pharmacists in the care of hospitalized patients has expanded over time, with increased emphasis on collaborative care and patient interaction. Pharmacy practice had to battle with a strict historic model of physicians diagnosing and prescribing while pharmacists compound and dispense. In a study, increased awareness of all team members’ (pharmacists, nurses and doctors) potential roles played a part in facilitating positive patient outcomes [35].

Fundamental to a good working relationship is when egos are set aside and the focus is set on preventing and solving medication errors with the intention of providing the best possible care for the patient.

### Study limitation

The length of time between when the pharmacist filed the clinical intervention report and the interview might have affected accuracy of recall by the pharmacists.

## Conclusion

The retrospective study findings clearly showed that clinical interventions formed a significant part of hospital pharmacists’ activities. These clinical interventions have been used to reduce the risk of medication errors in previous studies.

The barriers to undertaking clinical interventions related to workload, inadequate clinical knowledge and attitude of doctors and nurses. Although majority of pharmacists’ recommendations were accepted, participants mentioned that a major barrier was the attitude of doctors and nurses. Participants referred to the lack of confidence by these professionals in pharmacists’ clinical abilities. This led to lack of acceptance of some of their recommendations. Moreover pharmacists in this study perceived that doctors and nurses accepted more of their traditional roles and they appeared afraid of pharmacists taking over their clinical roles.
